# Dietary Copper Improves Intestinal Morphology via Modulating Intestinal Stem Cell Activity in Pigs

**DOI:** 10.3390/ani11092513

**Published:** 2021-08-26

**Authors:** Lanmei Yin, Qing Yang, Yiming Zhang, Dan Wan, Yuebang Yin, Qiye Wang, Jing Huang, Jianzhong Li, Huansheng Yang, Yulong Yin

**Affiliations:** 1 Laboratory of Animal Nutrition and Human Health, Hunan Provincial Key Laboratory of Animal Intestinal Function and Regulation, Hunan International Joint Laboratory of Animal Intestinal Ecology and Health, College of Life Sciences, Hunan Normal University, Changsha 410081, China; yinlanmei12@163.com (L.Y.); 15574980646@163.com (Q.Y.); 1347077364@163.com (Y.Z.); wangqiye@hunnu.edu.cn (Q.W.); jinghuang@foxmail.com (J.H.); ljzhong@hunnu.edu.cn (J.L.); yinyulong@isa.ac.cn (Y.Y.); 2 Hunan Provincial Key Laboratory of Animal Nutritional Physiology and Metabolic Process, Institute of Subtropical Agriculture, Chinese Academy of Sciences, Changsha 410125, China; w.dan@isa.ac.cn (D.W.); tonyerasmusyin@163.com (Y.Y.); 3 State Key Laboratory of Developmental Biology of Freshwater Fish, College of Life Sciences, Hunan Normal University, Changsha 410081, China

**Keywords:** copper, intestinal morphology, cell proliferation, intestinal stem cell, pig

## Abstract

**Simple Summary:**

Copper (Cu) is one of the essential trace elements for animal growth. Piglet intestinal epithelium undergoes a complete renewal driven by intestinal stem cells located at the crypt base. However, whether Cu improves intestinal morphology and development via modulating intestinal stem cell activity in pigs remains unclear. This study aimed to investigate the effects of dietary Cu during the weaning period on the intestinal morphology and development of weaned and finished pigs and explored the potential mechanism by culturing the intestinal organoids and treated them with Cu in vitro. The results indicated that Cu supplementation during the weaning period improves the intestinal morphology in finishing pigs by modulating the activity of intestinal stem cells.

**Abstract:**

Copper (Cu) is an essential micronutrient for animals. Many studies have been conducted on the effects of dietary Cu on growth performance, intestinal morphology, and function of piglets. However, the underlying mechanism remains to be explored. Intestinal stem cells (ISC) drive the development and constant renewal of intestinal epithelium. Therefore, we hypothesized that dietary Cu affects piglets’ intestinal development via modulating ISC activity. A total of eighty-five 21-day-old piglets were randomly assigned to five groups, where 25, 50, 75, 100, and 125 mg CuSO_4_/kg on a dry matter basis were supplemented to the basal diet at phase 1 (day 0 to 21). Increasing the dietary Cu concentration decreased (*p* < 0.05) villus width but increased (*p* < 0.001) the number of Ki67-positive cells. At phase 2 (day 22 to 163), the other 45 pigs were offered the same diets. Villus height in the 125 mg/kg Cu group was greater (*p* < 0.001) than in the other groups. Moreover, the effects of Cu on ISC activity in vitro were tested to explore the underlying mechanism. Compared to the control group, 10 μmol/L CuSO_4_·5H_2_O increased (*p* < 0.001) the organoid budding efficiency, crypt depth, and crypts per organoid. Dietary Cu improved the intestinal morphology of finishing pigs via promoting cell proliferation and modulating ISC activity.

## 1. Introduction

Copper (Cu) is an essential trace element for animal growth [1]. The dietary Cu requirement of weanling pigs is 5–6 mg/kg [2]. In commercial diets, 10–20 mg of inorganic Cu/kg is usually added [3]. Cu supplementation at pharmacological doses (150–250 mg/kg, resulted in piglets’ average body weight of 5.45 kg) improved growth performance and reduced the occurrence of diarrhea in weaned piglets [4,5]. Various studies have shown that high dietary Cu improves intestinal digestive and absorptive functions, which enhances growth performance, as the digestibility of energy, fat, and amino acids increases in pigs fed high Cu diets [6,7,8,9,10].

However, whether dietary Cu improves intestinal digestibility by affecting intestinal morphology is not clear because of the inconsistency of results. Dietary supplementation with 150 mg/kg of CuSO_4_ in the unprotected form increased duodenal villus height (VH) in weaned piglets [11]. However, 225 mg of CuSO_4_/kg in the diet had adverse effects on duodenal and jejunal VH in weaned piglets [12]. Radecki et al. found that 250 mg Cu/kg of dry matter did not affect intestinal the VH and crypt depth (CD) in weaned piglets [13]. Therefore, it still needs to be clarified whether Cu supplementation improves intestinal morphology in pigs. Moreover, intestinal morphology is dominated by the continual renewal of the epithelial cells along the crypt–villus axis [14]. It has been pointed out that dietary fiber increased goblet cells and altered the expression of the nutrient receptors and transporters to modulate intestinal epithelium differentiation in growing pigs, but it did not affect intestinal morphology [15]. Thus, dietary Cu may regulate cell renewal and morphology in pigs.

Cu is also required for the growth of various organs and the whole body of animals [16]. Intestinal stem cells (ISC) play a central role in modulating intestinal epithelium renewal and intestinal development [17]. Piglet intestinal epithelium undergoes a complete renewal every two to three days, which is driven by the ISC located at the crypt base [18]. Therefore, we hypothesize that dietary Cu affects intestinal development and functions via the ISC. The intestinal organoid is a newly developed technology that can simulate the characteristics of intestinal tissues, as it recapitulates in part the intestinal crypt–villus anatomy and can be used to detect the effects of substances on intestinal epithelial renewal and development [19]. Accordingly, the intestinal organoid is a good model for studying the effects of Cu on ISC activity in vitro. The present study was conducted to investigate how dietary Cu affects piglets’ intestinal morphology and development via modulating ISC activity.

## 2. Materials and Methods

The experimental protocol was reviewed and approved (Approval number 2016-093) by the Animal Care and Use Committee of Hunan Normal University.

### 2.1. Animals and Experimental Treatments

A total of eighty-five 21-day-old weaned piglets (Duroc × (Landrace × Yorkshire)) with an initial body weight of 4.98 ± 0.09 kg were randomly assigned to five dietary treatments. Previous studies have shown that dietary Cu affects the small intestine epithelium of weaned piglets offered the diets for two weeks, and the pigs were slaughtered for sampling at 100 kg during the finishing period [11,12,13,20]. Accordingly, the present trial lasted 163 days and included two phases: (day 1 to 21 and day 22 to 163). During phase 1, diets were supplemented with 25, 50, 75, 100, and 125 mg Cu/kg dry matter, respectively; the basal diets are displayed in Table 1. During phase 2, pigs from different treatments were given the same diet with Cu concentration according to NRC (2012) recommendations. The requirement of Cu for weaned piglets is 6 mg/kg according to the NRC (2012), thus, in the present experiment, percentage of Cu supplementation was 400%, 800%, 1200%, 1600%, and 2000%, relative to the Cu requirement indicated by the NRC (2012). All of the pigs were housed individually in a 1.2 × 0.5 m pen and were fed four times per day at 7:00, 11:00, 15:00, and 19:00. Feed and water were available to pigs throughout the experimental period. The room temperature was maintained at around 28–30 °C, and relative humidity was 60%.

### 2.2. Sample Collection

A total of eight randomly selected piglets from each treatment group with an average final body weight of 6.75 ± 0.18 kg were euthanized with 4% sodium pentobarbital solution for tissue sampling on day 21. Additionally, other pig group with an average body weight of 95.4 ± 1.9 kg were slaughtered by electrical stunning on d 163 according to standard commercial procedures [21]. After slaughtering, the small intestine was separated from the large intestine and was divided into three segments following the methodology of Wu et al. [22]. The duodenum was 10 cm from the pylorus, the distal ileum was 5 cm proximal to the ileocecal junctions, and the jejunum was separated from the middle segment. These segments were then rinsed with physiological saline. After removing the contents, the length and weight of the small intestine were measured. A 2 cm jejunal segment was fixed in 4% neutral-buffered formalin and was stored at room temperature before analysis.

### 2.3. Morphological Analysis

The jejunum sections were serially dehydrated with graded ethanol, cleared in xylene, and embedded in paraffin wax. Sections of 4-μm thickness were stained with hematoxylin and eosin and were examined under a light microscope (Leica DM3000; Wetzlar, Germany). Measurements were performed blindly using Image-Pro Plus 6.0 software (Media Cybernetics, San Diego, CA, USA), and the VH, CD, and villus width (VW) were measured [15,23]. Mean values of 15 fields, 30 well-oriented, complete villus-crypt structures were calculated for each pig.

### 2.4. Immunohistochemistry for Ki67

The slides were dried, dewaxed, rehydrated, and treated with 3% hydrogen peroxide (H_2_O_2_) in methanol for 10 min. Antigen retrieval was performed by boiling the slides twice in a sodium citrate buffer (0.01 mol/L, pH 6.0). A 5% bovine serum albumin (BSA; Boster Biological Technology Co. Ltd, Wuhan, China) was used in a 1:10 dilution during a 30 min incubation period at 37 °C. There was a two-hour incubation period with the Ki67 antibody (Abcam, ab15580; 1:800 dilution) at 37 °C. Sections were treated with a goat anti-rabbit IgG secondary antibody (ZSGB-BIO, Beijing, China) for one hour at 37 °C. With the exception of the blocking step, at every step, washing was completed thrice in PBS for 5 min. Positive cells were observed with a diaminobenzidine (DAB) Kit (ZSGB-BIO, Beijing, China), stained with hematoxylin, and made into permanent pieces. A total of 15 microscopic fields per sample were captured using a light microscope under 20× magnification (Leica DM3000, Leica Microsystems, Wetzlar, Germany). The number of Ki67-positive cells, at least 30 well-oriented complete crypts per sample was counted manually for analysis [24].

### 2.5. Cell Shedding Analysis

A total of thirty pictures per sample were captured using a light microscope under 10× magnification (Leica DM3000, Leica Microsystems, Wetzlar, Germany). The rate of cell shedding is expressed as the proportion of shedding villus in 100 intact villi [25,26].

### 2.6. Porcine Crypt Isolation, Organoid Culture, and Measurement

After euthanizing the piglets, the 3 cm segments of anterior jejunum best suited for organoid growth were removed and immediately rinsed with cold PBS to remove the intestinal contents. The fat and mesentery attached to the jejunum were removed with forceps and scissors. Then, the jejunum was dissected, cut longitudinally, and washed with ice-cold PBS. The mucosal surface was scraped with a glass coverslip to remove the villi [27]. The jejunum was then cut into small pieces (2–5 mm) and transferred to a 50 mL tube conical tube, and washed three times with ice-cold PBS. Epithelial isolation was performed by incubating the tissues in PBS supplemented with 2.5 mmol/L ethylenediaminetetraacetic acid disodium salt (Sigma-Aldrich, St. Louis, MO, USA) for 30 min at 4 °C on a rotator. Thereafter, the tissues were thoroughly suspended by being pipetted up and down 20 times with a 5 mL tip to loosen the crypts and were then filtered through a 70 μm cell strainer. An amount of 10% FBS was added to the crypt suspension and was spun down at 1200 rpm for 5 min. The supernatant was discarded, and the crypts were re-suspended in 2 mL complete medium, which consisted of advanced DMEM/F12 (Gibco, Grand Island, NE, USA), 1% of GlutaMAXTM Supplement (Gibco), 10 mmol/L of HEPES (Gibco), 100 U/mL of penicillin, 100 μg/mL streptomycin, and 1000 × antibiotic (Gibco). The crypts were then collected by means of centrifugation at 600 rpm for 5 min. Approximately 500 crypts were suspended in 40 μL cold Matrigel (Corning, Bedford, OH, USA). After that, one droplet of Matrigel/crypts mix was placed in the center of each well of a pre-warmed 24-well plate and was subsequently incubated at 37 °C with 5% CO_2_ for 15 min. A 500 μL amount of culture medium was added per well after the Matrigel was solidified. The culture medium was supplemented with Wnt3a, R-spondin 1, and Noggin (WRN) conditioned medium, FBS, N2 supplement (Gibco), B27 supplement (Gibco), n-acetyl-L-cysteine (Invitrogen, Carlsbad, CA, USA), nicotinamide (Sigma-Aldrich), epidermal growth factor (EGF, Sigma-Aldrich), A83-01 (TGF-beta inhibitor, Tocris, Bristol, UK), SB202190 (p38 inhibitor, R & D Systems, Minneapolis, MN, USA), 10 μmol/L Y27632 (Rho-kinase inhibitor, R & D Systems), and 2.5 μmol/L the glycogen synthase kinase 3 inhibitor (GSK3i, CHIR99021; Sigma-Aldrich). The culture medium was refreshed every two days, and the organoids were passaged every 6–7 days [28,29]. Li et al. treated porcine intestinal epithelial cells (IPEC-J2) with two doses (30 and 120 μmol/L) of CuSO_4_ [30]; therefore, the porcine organoid was incubated with 10 and 100 μmol/L CuSO_4_·5H_2_O (Sangon Biotech, A100330, Shanghai, China) for 72 h after passage. The organoid activity was determined by organoid budding efficiency, budding crypt depth, and crypts per organoid [31,32]. Organoid budding efficiency was calculated as a ratio of the budding organoid number to the total organoid number. Budding crypt depth was measured using Image-Pro Plus 6.0 software (Media Cybernetics, San Diego, CA, USA), while the number of crypts per organoid was expressed by counting the average number of buds.

### 2.7. Statistical Analysis

Data were analyzed using SPSS statistics 20 (SPSS Inc., Chicago, IL, USA). Before analysis, all of the data were tested for normality using histograms and the Shapiro–Wilk test. Any value that departed more than two standard deviations from the standardized mean was eliminated. One-way analysis of variance (ANOVA) was used if the data followed a normal distribution; otherwise, the Kruskal–Wallis test (VH, CD, and relative small intestine length at phase 1; CD at phase 2) was performed as the non-parametric testing. Values were expressed as means ± SEM. Individual piglets were the experimental units. Linear and quadratic contrasts of treatment were investigated. Differences among the treatments were examined using Duncan’s multiple comparisons, with *p* < 0.05 considered statistically significant and 0.05 < *p* < 0.10 considered as tending towards significance. All of figures in this study were drawn using Graphpad Prism 6.0 (GraphPad Inc., San Diego, CA, USA).

## 3. Results

### 3.1. Relative Small Intestinal Index

The small intestinal index of weaned and finishing pigs are shown in Table 2. Dietary Cu tended (ANOVA, *p* = 0.064; Liner, *p* = 0.026) to decrease the length of the small intestine. Dietary Cu did not affect the relative small intestine length and relative small intestine weight of either the weaned or finished pigs.

### 3.2. Intestinal Morphology

The gut morphology is presented in Table 3. On day 21 of age, no difference (*p* > 0.05) was observed in the VH. Moreover, the VW decreased (*p* = 0.037) with increases in dietary Cu supplementation. Offering 125 mg Cu/kg DM during the weaning period increased (*p* < 0.001) the jejunal VH in finished pigs.

### 3.3. Intestinal Epithelium Cell Proliferation and Cell Shedding

**Figure 1:**
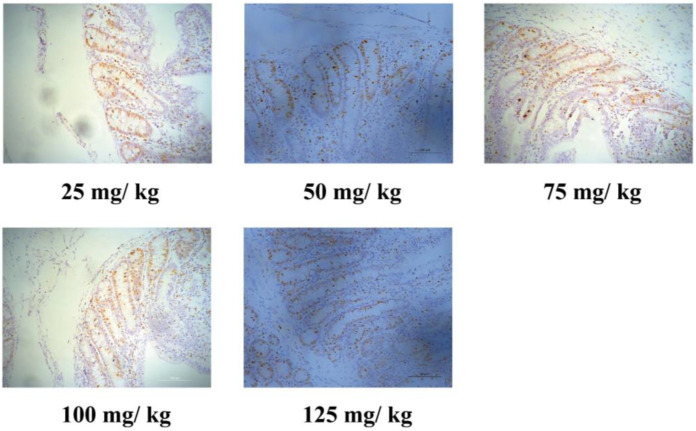
Representative immunohistochemical images of the Ki67-positive cells in the jejunum of weaned piglets. Pigs were supplemented with 25, 50, 75, 100, and 125 mg CuSO_4_/kg dry matter at the weaning phage (phase 1). Note Scale bars, 100 μm (magnification 200×).

Enterocyte proliferation and cell shedding are displayed in Table 4. The number of Ki67^+^ cells in the crypt increased (*p* < 0.001) with higher dietary Cu. Moreover, increasing dietary Cu tended to increase (quadratic, *p* = 0.060) the cell shedding rate. Representative images of the Ki67-positive cells are captured in Figure 1.

### 3.4. Jejunal Intestinal Organoid Activity

**Figure 2:**
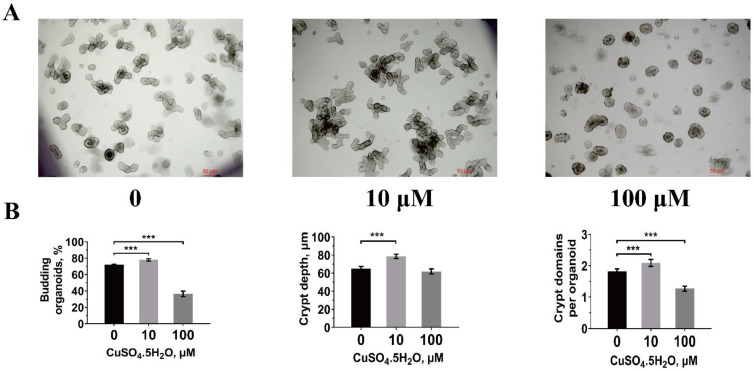
Effect of copper concentration on intestinal stem cells activity of piglets. (**A**) Representative images of organoid morphology after 0, 10, and 100 μmol/L CuSO_4_·5H_2_O treatment in vitro. Scale bars, 50 μm (magnification 50×). (**B**) Organoid budding efficiency, budding crypt depth, and crypts per organoid at three days after CuSO_4_·5H_2_O treatments were quantified. Statistical significance was determined by one-way ANOVA. The results are expressed as the means and SEM (*n* = 6 wells per treatment; *** *p* < 0.001).

In vitro intestinal stem cell activity obtained from piglets treated with 10 and 100 μmol/L CuSO_4_·5H_2_O is presented in Figure 2. We found that 10 μmol/L CuSO_4_·5H_2_O significantly increased (*p* < 0.001) organoid budding efficiency, crypt depth, and crypts per organoid compared to the control pigs (Figure 2B). However, 100 μmol/L CuSO_4_·5H_2_O inhibited (*p* < 0.001) the organoid budding efficiency and the crypts per organoid.

## 4. Discussion

Small intestine morphology, including VH, CD, and their ratio, have commonly been used as indicators of piglets’ intestinal health [33]. Giancamillo et al. showed that dietary Cu could increase intestinal VH in weaned piglets, which is consistent with our results [11]. The intestinal epithelium is composed of a monolayer of epithelial cells that undergo continual renewal along the crypt–villus axis, and VH and epithelial cell populations are highly correlated [14]. Changes in intestinal morphology are usually accompanied by the process of epithelial cell renewal [34]. Our previous study showed a positive relationship between cell proliferation and jejunal VH and CD in weaned piglets [24]. To determine whether the changes in intestinal morphology resulted from the alteration in epithelium renewal by dietary Cu, we counted Ki67-positive proliferating cells to the crypt base and cell shedding rates at the villus tips in weaned piglets. The number of Ki67^+^ cells in the crypt increased with increasing the amount of dietary Cu, which indicated that high Cu ingestion increased intestinal epithelial cell proliferation. Ki67, a marker for proliferating cells, labeled the undifferentiated proliferating transit-amplifying (TA) cells (progenitors) at the crypt, which stem from the ISC and differentiated into functional epithelial cells [35,36,37]. Moreover, increasing the dietary Cu tended to increase the cell shedding rates. These results indicated that dietary Cu might modulate the ISC to increase cell proliferation and affect intestinal cell renewal in weaned piglets, which may regulate intestinal morphology. Length and weight are primary indices of intestinal development, which are highly related to intestinal digestion and absorption capacity [38,39]. Dietary Cu supplementation only tended to decrease the length of the small intestine and had no effects on the relative small intestinal index of the weaned piglets, which indicates that dietary Cu has minor effects on the intestinal development of weaned piglets.

The weaning period is an important developmental window for the small intestine of animals, and any effects on their developmental programming during early life, especially during the developmental windows, will have lifelong implications [40,41]. Previous studies tested the effects of dietary Cu during the nursery or finishing period, but no experiments had tested the effects of dietary Cu during early life on the intestinal development of the finishing period. The results of the present study showed that dietary supplementation with 125 mg Cu/kg DM during the weaning period increased jejunal VH in finishing pigs at 163 d of age. Dietary Cu did not affect the relative small intestine length or the relative small intestine weight. These results indicate that dietary Cu during the weaning period improves the intestinal morphology in finishing pigs.

Piglet intestinal epithelium undergoes a complete renewal every two to three days, which is driven by the ISC located at the crypt base, which first generate transit-amplifying (TA) cells that further differentiate into absorptive (enterocytes) or secretory cell lineages (Paneth cells, goblet cells, and enteroendocrine cells) [18]. Enteroids cultured from ISC partially recreate the villus-crypt anatomy of the native intestine and mimic hallmarks of in vivo epithelium [42]. The ISC play a crucial role in maintaining gut morphology and intestinal development [17]. To test whether the effects of dietary Cu on intestinal morphology and development acted through the ISC, the jejunal organoids from 21-day-old piglets were treated with different concentrations of CuSO_4_·5H_2_O. Organoid budding may be similar to the expansion of the intestinal stem cell compartment and the formation of new crypts through crypt fission [43]. A greater crypt depth in the organoids suggests increased stem cell proliferation and differentiation, which resembles the increase in cell renewal along the crypt–villus axis in the intestinal mucosa [44]. We found that dietary Cu stimulated the epithelial proliferation with increased Ki67^+^ cells at the crypt in vivo, and enteroid growth with increased budding efficiency, crypt depth, and crypts per organoid under Cu exposure in vitro. Wang et al. indicated that enterocyte proliferation was positively related to mucosal enzyme activities and nutrient digestibility [24]; whether dietary Cu improves intestinal morphology by altering the expression of nutrient receptors and transporters to modulate intestinal differentiation in growing pigs needs to be further explored, although this was demonstrated by Saqui-Salces et al. in study on fiber [15]. These results indicate that Cu modulates the activity of the ISC and that dietary Cu may, by increasing intestinal epithelial cell proliferation and impacting the ISC, improve intestinal morphology in finishing pigs. Unfortunately, the commercially available antibodies used to detect the intestinal stem cells, Lgr5, did not demonstrate cross-reactivity with the active CBC stem cells in the porcine jejunal tissue. Further experiments are needed to test the potential mechanism of Cu to modulate ISC activity in vitro.

## 5. Conclusions

Dietary Cu supplementation during the weaning period improved intestinal morphology of finishing pigs, which may be achieved by promoting cell proliferation and increasing the activity of ISC.

## Figures and Tables

**Table 1:** Basal diet ingredient and nutritional component (as-fed basis).

Ingredients	Content (%)
Corn grain	37.16
Extruded corn	20.00
Soybean meal (43% CP)	8.00
Soy protein concentrate powder	7.00
Whey powder	10.00
Fish meal (63% CP)	5.00
Spray-dried plasma protein	4.50
Glucose	2.00
Soybean oil	2.00
Vitamin and mineral premix ^a^	4.34
Total	100.00
Calculated nutrient levels	
Crude protein, %	19.00
ME, MJ/kg	13.25
NDF, %	6.40
ADF, %	2.30
Calcium, %	0.75
Available phosphorous, %	0.38
Lysine, %	1.38
Metionine, %	0.40
Met + Cys, %	0.80
Threonine, %	0.86
Triptophane, %	0.25
Cu, mg/kg	5.04

^a^ Vitamin-mineral premix supplied per kilogram of feed: Vitamin A: 2200 IU; Vitamin D_3_: 220 IU; Vitamin E: 16 IU; Vitamin K_3_: 0.5 mg; Vitamin B_12_: 0.0175 mg; Riboflavin: 3.5 mg; Niacin: 30 mg; D-pantothenic acid: 10 mg; Biotin: 0.05 mg; Folic acid: 0.3 mg; Thiamine: 1.0 mg; FeSO_4_: 150 mg; ZnSO_4_: 100 mg; MnSO_4_: 30 mg; KIO_3_: 0.5 mg; CoSO_4_: 0.3 mg; Na_2_SeO_3_: 0.3 mg; Ethoxyquin: 4.0 mg.

**Table 2:** Effects of dietary copper supplementation during the weaning period on the small intestinal index of weaned and finished pigs ^1^.

Variables	Dietary Copper, mg/kg Dry Matter					SEM ^4^	*p*-Value		
	25	50	75	100	125		ANOVA	Linear	Quadratic
Phase 1 (day 1 to 21) ^2^									
Small intestine length, m	12.4	12.5	11.5	11.7	11.8	0.83	0.064	0.026	0.244
Small intestine weight, g	516.3	537.7	494.6	529.8	478.4	55.84	0.190	0.179	0.355
Relative small intestine length, m/kg	1.6	1.9	1.7	1.8	1.8	0.06	0.464	0.402	0.624
Relative small intestine weight, g/kg	71.0	83.1	76.4	76.8	74.5	2.41	0.705	0.965	0.309
Phase 2 (day 22 to 163) ^3^									
Small intestine length, m	19.0	18.6	19.0	18.3	18.7	0.24	0.897	0.624	0.671
Small intestine weight, kg	1.7	1.7	1.8	1.6	1.7	0.04	0.768	0.521	0.573
Relative small intestine length, m/kg	18.9	20.3	20.2	19.1	20.9	0.34	0.296	0.259	0.987
Relative small intestine weight, kg/kg	16.8	18.6	18.9	16.9	18.3	0.35	0.198	0.551	0.306

^1^ Pigs were supplemented with 25, 50, 75, 100, 125 mg CuSO_4_/kg dry matter at the weaning phage (phase 1), and all pigs from different treatments were given the same diet with Cu concertation according to the NRC (2012) recommendations for finishing pigs (phase 2). ^2^ *n* = 8. ^3^ *n* = 9. ^4^ SEM, pooled standard error of the mean. Relative small intestine weight = the ratio of total small intestine weight to body weight at slaughter; Relative small intestine length = the ratio of total small intestine length to body weight at slaughter.

**Table 3:** Effects of dietary copper supplementation during the weaning period on intestinal morphology of weaned and finished pigs ^1^.

Variables	Dietary Copper, mg/kg DM					SEM ^4^	*p*-Value		
	25	50	75	100	125		ANOVA	Linear	Quadratic
Phase 1 (day 1 to 21) ^2^									
Villus height, μm	305.9	359.5	303.5	333.4	346.0	9.30	0.160	0.413	0.959
Crypt depth, μm	319.6	324.5	327.4	321.6	296.0	10.39	0.753	0.521	0.449
Villus height: crypt depth, μm:μm	1.0	1.2	1.0	1.1	1.1	0.05	0.646	0.587	0.864
Villus width, μm	175.9 ^a^	146.0 ^bc^	153.7 ^bc^	161.3 ^ac^	147.7 ^bc^	3.42	0.037	0.075	0.226
Intestinal villus surface area, mm^2^	0.2	0.2	0.1	0.2	0.2	0.01	0.598	0.706	0.471
Phase 2 (day 22 to 163) ^3^									
Villus height, μm	409.7 ^b^	379.1 ^b^	393.8 ^b^	368.6 ^b^	512.1 ^a^	13.83	<0.001	0.016	0.002
Crypt depth, μm	443.1	402.5	414.9	407.6	467.3	9.93	0.158	0.424	0.030
Villus height:crypt depth, μm:μm	0.9	0.9	1.0	0.9	1.1	0.03	0.245	0.175	0.368

^1^ Pigs were supplemented with 25, 50, 75, 100, 125 mg CuSO4/kg dry matter at the weaning phase (phase 1), and all pigs from different treatments were given the same diet with Cu concentration according to the NRC (2012) recommendations at the finishing phase (phase 2). Intestinal morphology was measured in the jejunum. ^2^ *n* = 8. ^3^ *n* = 9. ^4^ SEM, pooled standard error of the mean. ^a, b, c^ Within rows, means labeled with different superscripts differ (*p* < 0.05). Intestinal villus surface area = π * villus height * villus width.

**Table 4:** Effects of dietary copper supplementation on intestinal epithelium renewal of weaned pigs ^1^.

Variables	Dietary Copper, mg/kg DM					SEM ^2^	*p*-Value		
	25	50	75	100	125		ANOVA	Linear	Quadratic
Ki67-positive cells per crypt, *n*	22.3 ^b^	27.4 ^a^	25.5 ^ac^	24.6 ^bc^	27.7 ^ad^	0.50	<0.001	0.007	0.381
Cell shedding rate, %	25.4	29.1	28.8	28.4	28.3	0.48	0.134	0.135	0.060

^1^ Pigs were supplemented with 25, 50, 75, 100, 125 mg CuSO_4_/kg dry matter. ^2^ SEM, pooled standard error of the mean (*n* = 8). ^a, b, c, d^ Within rows, means labeled with different superscripts differ (*p* < 0.05).

## Data Availability

All data are available from the corresponding author on reasonable request.
